# A survey in Austria supports the significance of genetic counseling and pharmacogenetic testing for mental illness

**DOI:** 10.3389/fpsyt.2024.1436875

**Published:** 2024-10-03

**Authors:** Elena Aschauer, Shahriar Izadi Yazdi, Harald Aschauer

**Affiliations:** ^1^ Allgemein Psychiatrische Abteilung, Klinik Landstrasse, Wiener Gesundheitsverbund, Vienna, Austria; ^2^ Erste Psychiatrische Abteilung mit Zentrum für Psychotherapie und Psychosomatik, Klinik Penzing, Wiener Gesundheitsverbund, Vienna, Austria; ^3^ Biopsychosocial Corporation, BioPsyc, Non-profit Association for Research Funding Ltd, Vienna, Austria

**Keywords:** genetic counseling, genetic testing, mental illness, survey, general population, experts, attitude, interest

## Abstract

**Introduction:**

Genetic counseling and testing in psychiatry warrant attention, but research results on attitude, knowledge, personal experience and interest are limited. There are only a few studies that have compared the opinions of the general population and experts regarding genetic counseling and genetic testing in mental illness.

**Methods:**

This study aimed to investigate these gaps through a cross-sectional survey conducted in Austria, involving a sample of the web-active population, representative according to gender, age and geographical location (n=1,000, 24.5% of them had a psychiatric diagnosis), and experts (n=145, 83.4% of them psychiatrists). Two questionnaires were developed. Pearson chi-square statistics were used to compare responses, and regression analyses were employed to measure the strength of psycho-sociodemographic influences on answers.

**Results:**

The findings revealed that public considered genetic counseling to be more important than experts did (68.8% versus 54.2%; Pearson chi-square 12.183; df=1; p<0.001). The general population believed that genetic testing is useful for diagnosing mental disorders, which contrasted with experts’ opinions (67.9% versus 17.2%; Pearson chi-square 137.236; df=1; p<0.001). Both groups agreed on the potential benefits of pharmacogenetic testing (79% versus 80%). A small number of individuals from the public had sought genetic counseling (8%), and only a minority of experts had specific training and experience in this field (28%).

**Discussion:**

This is the first survey study on the topic conducted in Austria, with limited international studies available. Austrian experts place less value on genetic counseling compared to their counterparts in other countries. Despite recognized importance placed on genetic counseling and testing, utilization rates remain low. The value of pharmacogenetics is predicted to increase in the future. Consequently, it is crucial for medical training programs to emphasize the significance of genetic counseling and enhance the understanding of genetic aspects related to mental illnesses to enable experts to provide adequate psychoeducation and personalized care to the extent possible to patients and their families.

## 1 Introduction

Mental disorders are common. According to a world-wide population survey, the lifetime prevalence of being diagnosed with any psychiatric disorder is 28.6% for males and 29.8% for females (1). The most common disorders in males are any anxiety disorder (11.3%), any mood disorder (9.5%), and any substance use disorder (15.6%), whereas in females, the lifetime prevalence is 18.8%, 15.4% and 4.5%, respectively. It is well known that mental disorders run in families (2). Estimates of twin heritability are high, range from around 37% for Major Depressive Disorder to around 81% for Schizophrenia (3). However, estimated SNP- heritability is much lower, ranging from 5.3% for posttraumatic stress disorder to 29.3% for obsessive compulsive disorder (4, 5). Despite substantial progress in the last decade, the multifactorial genetic causal factors mostly remain elusive. Some large-scale studies have discovered an increasing number of common genetic variants with low penetrance associated with mental disorders, exhibiting high polygenicity and genetic overlap between disorders (4, 6, 7). Additionally, we are learning more about the influence of rare variations with higher penetrance, most notably demonstrated by “copy number variations” (CNVs).

Genetic counseling is a specialized medical service provided over multiple sessions. Professionals providing genetic counseling interpret and explain genetic information to patients and support them throughout the process. They counsel patients regarding decisions about genetic testing, can order genetic tests, discuss the results, calculate genetic risks, and explain inheritance patterns. They also manage the psychosocial and psychological consequences for individuals and their families (8). Genetic counseling can be applied to all conditions with a genetic component, making it a valuable asset in psychiatric care. It helps reduce stigma, enhances understanding and classification of disorders within families, and improves the management of these disorders. Meta-analyses have demonstrated positive outcomes associated with genetic counseling (9–13). In both pre- and post-genetic testing scenarios, genetic counseling plays a critical role (14). In Austria, genetic counseling and testing are regulated by the Genetic Engineering Act (15). Genetic counseling in Austria is exclusively provided by medical professionals. In many countries worldwide, the profession of a “genetic counselor” has long been established as an integral component of genetic testing services (16, 17).

Despite considerable progress in the field of psychiatric genetics in recent years, yielding many significant and replicable findings, validated genetic tests for predicting a mental disorder in pre-symptomatic individuals and diagnosing common mental disorders in symptomatic patients are still lacking. The International Society of Psychiatric Genetics (ISPG), as the preeminent professional society in this field, periodically updates a policy paper on its website to provide guidance for physicians (18). In certain circumstances, diagnostic genetic testing can be warranted. For example, CNVs and single-gene variants with large effects, which are rare genetic changes in DNA (“deoxyribonucleic acid”), are strongly linked to mental disorders such as intellectual disability (ID) and autism spectrum disorders (ASD). Some genetic tests for CNVs have become part of routine clinical genetic assessments for children with “neurodevelopmental disorders”, such as ID (19–21). However, this is not yet the case for ASD and schizophrenia (22). Attitudes, barriers and beliefs regarding genetic testing vary among patients, experts, and the public, even when it comes to organic mental disorders like dementia (23, 24). Studies have shown substantial interest among patients and their families in diagnostic and predictive genetic testing in psychiatry. Some psychiatrists also share this interest (25).

Pharmacogenetic testing provides information about genes to help choose the most suitable medicine and dosage for individual patient. It is gaining traction in the field of psychiatry (26–31). There are two branches of pharmacology: pharmacokinetics, which refers to the path of a drug into, through and out of the body (drug metabolism), and pharmacodynamics, which concerns the action of drugs on the body. In some countries, there are legal recommendations, though not requirements, for genetic testing before prescribing certain psychotropic drugs to protect individuals from severe adverse drug reactions (32). This is particularly relevant for genetic testing related to specific polymorphisms in the human leukocyte antigen system (HLA). Certain HLA types can predispose carriers to life-threatening skin conditions, such as Stevens-Johnson syndrome, when exposed to drugs like carbamazepine. Over the past two decades, research has identified various genes that influence drug metabolism. One crucial family of enzymes central to drug metabolism is Cytochrome P450 (CYP). Genetic variations in genes encoding specific members of this family, such as enzymes CYP2D6 and CYP2C19, result in differences in the metabolism of many drugs used in psychiatry, especially antidepressants and antipsychotics (33). Patients with certain variants are at an increased risk of experiencing adverse drug reactions, while patients with other variants may be less responsive to treatment (26, 34). While there is sufficient scientific support for clinical recommendations for some psychotropic drugs based on variants in pharmacokinetic genes, knowledge about how neurotransmitter receptor genes influence the therapeutic response to psychiatric medications is not yet well-established enough to predict treatment outcomes through genetic testing before therapy initiation. Currently, intense research efforts are underway to deepen the knowledge of pharmacogenetics in psychiatry to provide more personalized care to patients (35). Clinical studies suggest that testing may be most beneficial for individuals who have previously experienced an adverse drug reaction or inadequate response to medications (18, 27, 36, 37). Detailed information about pharmacogenetic findings, including dosing recommendations, is collected and published by the Clinical Pharmacogenetic Implementation Consortium (CPIC) (27) and the Dutch Pharmacogenetics Working Group (DPWG) (38).

There are only a few studies that have compared the opinions of the general population and experts regarding genetic counseling and genetic testing in mental illness. Some of these studies focused on specific mental illnesses, while others have centered on patients and their relatives. It’s also important to differentiate between surveys that yield quantitative results and those that provide qualitative insights.

A qualitative study conducted in the United Kingdom (UK) on genetic counseling in psychiatry gathered data from 32 health professionals (39). At that time, genetic counseling was not routinely offered for psychiatric disorders in UK. However, the healthcare professionals believed that genetic counseling would be valuable and desirable. Another qualitative survey was conducted in United States (USA), Colorado, involving twenty patients with depression who had previously undergone psychiatric pharmacogenetic testing due to failed therapies or medication intolerance (40). The experiences ranged from optimism to disappointment. In Canada, researchers published the results of a qualitative survey involving patients (n=7) and experts (n=15) on pharmacogenomic testing for depression. Both groups expressed a need for more evidence and had concerns, particularly related to economic issues and subject-specific training (41). In the UK, scientists conducted a qualitative survey in 14 genetics practitioners, resulting in several key findings: a need for increased workforce capacity, enhanced access to psychological support for patients, and more specialized knowledge in psychiatric genetics (42).

Quantitative surveys available in the literature will be reported alongside our data in discussion.

Currently, there is no available information on scientific studies and publications on the attitudes, knowledge, personal experiences, and interest to genetic counseling and testing for mental disorders among the public and experts in Austria. However, obtaining such information is crucial to developing an adapted healthcare plan. There is a significant need for research in this area. This study was conducted to address among others the following questions concerning mental disorders among the public and experts in Austria, and to compare responses between them: How useful do the groups consider genetic counseling and testing for diagnosing mental disorders? Do the groups believe genetic tests are currently available for diagnostic purposes? Furthermore, the study aims to investigate the influence and extent of psycho-sociodemographic factors on the responses to the following questions: Do individuals from the general population and experts perceive pharmacogenetic testing as useful? How many people have utilized genetic counseling and testing, and how many experts have employed these services? Have experts undergone specialized training in genetic counseling? How willing are individuals to undergo genetic counseling, genetic testing and pharmacogenetic testing?

## 2 Methods

This publication is founded on a master’s thesis that delved into the economic viability of establishing a medical practice for genetic counseling in psychiatry (43). While the methods and raw data values of the surveys were initially presented in German within the thesis, the specific findings and statistics presented here are being published for the first time.

### 2.1 Study design, survey development, questionnaires and pre-testing

Cross-sectional surveys were conducted in Austria, targeting both the representative general population (referred to as the ‘public’) and experts. These surveys were administered by a reputable market research firm, “Das Österreichische Gallup Institut” (www.gallup.at);. The survey design targeted to collect personal insights of public and experts regardless the level, quality and source of their prior knowledge on genetic counseling and testing in general and in psychiatry.

To gather data, two distinct questionnaires in German with closed questions were developed exclusively for this study, validated and administered (questions in detail can be found in Appendix 1 and 2, translations of the questions into English were produced to be shown here and can be found in Appendix 3 and 4). These questionnaires were designed by the authors based on a thorough review of relevant existing literature (references see in discussion), clinical expertise of authors (psychiatry, neurology, psychotherapy, human genetics, psychiatric genetic counseling) and adaptions to the local context in Austria. The first questionnaire, consisting of 35 items, was intended for use with adult participants from the public. The second, comprising 36 items, was tailored for experts. Both questionnaires were structured around four key domains, all in psychiatric counseling and diagnostic- and pharmaco-genetic testing: attitude, knowledge, personal experience, and interest. Additionally, they included items measuring socio-demographic variables (age, gender) and questions pertaining to personal histories of psychiatric disorders, profession, education, place and population of residence, household income within the public, and questions related to specialization, training and education and workplace within experts. Information regarding the purpose of the study and how the data would be used was given at the beginning of the survey. Respondents were told that it is not about them having concrete knowledge of certain things, but about their personal, emotional assessment, experience and intention. Responding to each questionnaire typically required 10 to 15 minutes. The questions in these surveys took two main formats: either dichotomous (‘yes’ or ‘no’) or employing a Likert-scale ranging from 1 (‘very useful´, ´very satisfactory´ or ‘most likely’) to 5 (‘not useful at all’, ´not at all satisfactory´ or ‘not likely at all’). First versions of both questionnaires were pre-tested to ensure ease of use and appropriateness of the survey content among five individuals from the public and five experts respectively, all drawn from the authors’ network. These individuals provided feedback on the draft, leading to minor revisions focused on clarifying certain items, simplifying questions and adding some explanations. The second versions were again reviewed by the experts as before. After minor revisions again, psychologists of the market research company, experienced in survey methods, conducted a review, leveraging their expertise in content conception, formatting, testing, and filter implementation. Where necessary, explanations for specific terms were incorporated. The questionnaires were accordingly revised to their third versions. Subsequently, the authors conducted a final review, and the questionnaires were prepared for administration. The nine questions of the questionnaire for the public, that were statistically evaluated in the current analyses, were subjected to a test-retest-reliability test on a representative sample of 150 individuals from the web-active population in Austria aged 18 years and older. This sample from the online access panel (see recruitment below) was stratified according to specific quotas (gender, age, education – with or without Matura). The second wave of test-retest surveys took place approximately ten days after the first. In the first wave a corresponding excess of interviews were carried out, so that a base of 150 interviews was created in the second wave. Only people who took part in both waves were, of course, included in the test-retest-reliability study. An incentive was given even if somebody only took part in the first wave of the survey.

### 2.2 Participants, recruitment, data collection, quality check, sample size

#### 2.2.1 General population (public)

The survey of Austria’s adult population was conducted among individuals who were representative of the web-active population aged 18 years and older (see **Table 1**). Every person in the population has the same chance of becoming part of the sample. The sampling is two-stage: in the first step, panel participants are recruited from the population, in the second step, the sample is drawn from the panel. This sample was stratified according to specific quotas (gender, age, and federal state of residence, ´random quota method´) and it comprised 1,000 participants (245 of them had a psychiatric diagnosis). The quotation and subsequent weighting (´random iterative method´) ensured that the sample represents a representation of the population in terms of important structural characteristics. Incentives were provided to panelists. Data collection for this sample took place between October 20 and 23, 2020. The online access panel used for this survey adheres to stringent quality criteria, including measures to prevent false or multiple registrations through methods such as manual screening and automated verification algorithms. Additionally, participant authentication is based on bank details, and a double opt-in procedure is in place. The panel maintains comprehensive profile data, which allows for precise participant selection. Furthermore, the company strictly adheres to regulations designed to protect the data and privacy of panelists.

**Table 1:** Structure of respondents from the general population (public) (43).

	Population: number (%)
**Total**	1,000 (100)
Gender	
Male	488 (48.8)
Female	512 (51.2)
Age (years)	
18 – 30	200 (20.0)
31 – 50	342 (34.2)
>50	458 (45.8)
Federal state of residence	
Vienna	213 (21.3)
Lower Austria, Burgenland	224 (22.4)
Styria, Carinthia	208 (20.8)
Upper Austria, Salzburg	227 (22.7)
Tyrol, Vorarlberg	128 (12.8)
Profession	
Self-employed, liberal profession, managerial staff	65 (6.5)
Civil servants, employees	330 (33.0)
Workers	143 (14.3)
Pupil, student	52 (5.2)
Not employed	140 (14.0)
Pensioner	270 (27.0)
Highest educational degree	
No qualifications, compulsory education	195 (19.5)
Vocational or technical school without Matura	486 (48.6)
Matura, technical college, university	319 (31.9)
Mental illness*	
Yes	245 (24.5)
No	755 (75.5)

*The following question was asked: “Have you ever been diagnosed with a mental illness?”

#### 2.2.2 Expert sample

It comprised a total of 145 participants (see **Table 2**), divided into two groups. The first group consisted of medical doctors, all in psychiatry, from across Austria (n=94). The second group consisted of experts from diverse educational backgrounds (n=51, 47.1% of them psychiatrists). For the 51 experts, we conducted interviews using Computer Assisted Telephone Interviews (CATI) with the assistance of two specially trained senior study leaders from the market research firm having experience with target groups from the medical field. A search process was started to collect the eligible sample, after which an attempt was made to have contact by telephone. If the experts were generally willing to participate, an appointment was made for the interview. Incentives were offered. Quality measures of interviews and supervision were also carried out during CATI (checks, listening, plausibility by looking at the statistics per interviewer, telephone follow-up to check the participation of the respondents). On the other hand, the 94 medical doctors completed an online questionnaire through Computer Assisted Web Interviews (CAWI). Each doctor received a personalized questionnaire link after expressing willingness to participate, with each link usable only once and by a single individual. No specific quotas were applied to the expert sample. Data collection for this sample took place from October 27 to December 8, 2020.

**Table 2:** Structure of respondents from experts in Austria (43).

	Experts: number (%)
**Total**	145 (100)
Gender	
Male	74 (51)
Female	71 (49)
Age (years)	
<35	12 (8)
35 – 55	90 (62)
>55	43 (30)
Completed medical specialist training or other education*, **	
Psychiatry and psychotherapeutic medicine	85
Psychiatry	38
Psychiatry and neurology	22
Child and adolescent neuropsychiatry and psychotherapeutic medicine	10
Neurology	8
Neurology and psychiatry	8
Child and adolescent neuropsychiatry	7
Human genetics	3
Medical genetics	2
Other education	19
Current job or work setting**	
Hospital	90
Medical ordination as “Wahlarzt” (elective doctor, whose bill gets fully or in part paid by health insurance in Austria)	75
Psychosocial service	15
Medical ordination with health insurance contracts	10
Other facility	14

*Medical specialist training according to training regulations of Austrian Medical Association.

**Multiple entries possible.

During the sampling process, the company conducted a quality check to ensure that all interviews met the criteria for inclusion in the analysis. Individual information was compared with each other and checked for plausibility (e.g. the variables of town size and federal state or occupation and education with age). Furthermore, the average interview length was determined in order to eliminate interviews that were too short. It was also checked whether there was obviously random response behavior and the proportion of ´no information´ answers should not be too high per interview. All interviews met the quality standards, and the data were stored anonymously.

The total population of Austria is approximately 8,900,000 (December 2020). In our representative survey, we included a sample of 1,000 individuals. With this number of cases a fluctuation range of +/- 1.4 to +/-3.2 is assumed. According to statistics from the Austrian Medical Association as of December 31, 2020 (44), Austria had approximately 4,000 specialists in related fields (e.g., psychiatry and psychotherapeutic medicine, neurology, child and adolescent psychiatry). Our expert sample consisted of 145 participants.

In the population survey, one of the questions asked was, “Have you ever been diagnosed with a mental illness?” The responses indicated that 24.5% of respondents had previously received a diagnosis of a mental disorder. Furthermore, we inquired about the specific mental disorder diagnosed in individuals who had reported a prior diagnosis. Respondents were presented with a multiple-choice question listing psychiatric diagnoses according to ICD-10 (45). The most common mental disorders identified in our survey were mood disorders (71%), anxiety disorders (33%), substance use disorders (including alcohol, 12%), posttraumatic stress disorder (12%) and bipolar disorder (9%).

### 2.3 Statistics

Descriptive statistics, such as numbers and percentages, were employed to characterize the samples, including demographics (refer to **Tables 1**, **2**). For Likert-scale questions, in some statistics, responses were recoded into a dichotomous variable (responses 1 and 2 were categorized as agreement, to summarize the answers with strong agreement, while responses 3 to 5 indicated disagreement). As an exploratory study, correlations with p-values less than 0.05 were considered suggestive of statistical significance, and no multiple testing correction was applied. The presented results are based on the analysis of a subset of questions. The exact wording of the questions is available in Appendix 1 and 2 and in **Tables 3**-**5**. We examined responses to three questions that were posed to both the public and experts, enabling a statistical comparison, focusing on attitudes and knowledge, using Pearson chi-square statistics and p-values (see **Table 3**). Additionally, we analyzed questions specific to each group, which were not suitable for direct comparison but were utilized in regression analysis, covering attitudes, knowledge, personal experience and interest. Linear or logistic regression analyses were conducted to determine the extent of psycho-sociodemographic influences (independent variables) on the responses, utilizing various statistical tests including ANOVA, chi-square, omnibus test, Wald test, and associated p-values. In the public, four parameters were tested as potential independent variables: age (a metric variable), gender (coded as 0 for ‘men’ and 1 for ‘women’), highest educational degree (coded as 0 for ‘no qualifications, compulsory education, vocational or technical school without Matura’ and 1 for ‘Matura, technical college, university’), and mental illness status (a binary variable, coded as 0 for ‘yes’ and 1 for ‘no’, in response to the question ‘Have you ever been diagnosed with a mental illness?’ – see **Table 1**). Among experts, the influence of age and gender on responses was examined (refer to **Tables 4**, **5**). Three individuals from the general population did not provide their exact age and were consequently excluded from regression analyses. Statistical analysis was conducted using IBM SPSS version 22.

**Table 3:** Comparison of responses from the general population (public) and experts to questions (see **Appendix 1-4 in Supplementary Material**) about attitudes and knowledge regarding genetic counseling and genetic testing.

Questions					
		Approval: number (%)	Rejection: number (%)	Pearson chi-square (df=1)	Asympt. sign. (2-sided)
Attitude					
Qu. 3 (public) and qu. 1 (experts): “How useful do you think genetic counseling is for mental disorders?” (Likert-scale)					
	General population	688 (68.8)	312 (31.2)		
	Experts*	78 (54.2)	66 (45.8)		
				12.183	p<0.001
Qu. 25 (public) and qu. 20 (experts): “How useful do you think genetic testing is for determining a diagnosis in the case of mental disorders?” (Likert-scale)					
	General population	679 (67.9)	321 (32.1)		
	Experts	25 (17.2)	120 (82.8)		
				137.236	p<0.001
Knowledge					
Qu. 23 (public): “In your opinion, are genetic tests currently available that make it possible to diagnose mental disorders?” (dichotomous)					
	General population**	262 (26.2)	735 (73.8)		
Qu. 14 (experts): “Are genetic tests currently available, the use of which enables the diagnosis of mental disorders?” (dichotomous)					
	Experts***	49 (35.8)	88 (64.2)		
				5.447	p=0.020

*Missing value: 1.

**Missing values: 3.

***Missing values: 8.

**Table 4:** Questions (see **Appendix 1-4 in Supplementary Material**) and answers about knowledge and interest regarding genetic counseling and testing among the general population (public) and experts.

Question					
		Approval: number (%)	Rejection: number (%)	Logistic regression	sign.
Knowledge					
Qu. 12: “Have you undergone special training in genetic counseling (in Austria or abroad)?” (dichotomous)	Experts	41 (28)	104 (72)	omnibus test chi-square 18.033, df=2	p<0.001*
Interest					
Qu. 5: “Would you get genetic counseling if you needed it (because of a mental disorder)?” (dichotomous)	Public	790 (79)	210 (21)	omnibus test chi-square 2.355, df=4	p=0.671
Qu. 13: “If you were offered a genetic test, you would do it?” (dichotomous)	Public	740 (74)	260 (26)	omnibus test chi-square 15.081, df=4	p=0.005**
Qu. 32: “How likely is it that you will be genetically tested if you do not respond to drug therapy?” (Likert-scale)	Public	510 (51)	490 (49)	F=0.117, df=4	p=0.976

*Older experts were more likely to have received training in genetic counseling.

**Likelihood of agreeing to genetic testing increased among individuals who had already received a psychiatric diagnosis.

The influence of psycho-sociodemographic factors on the answering was calculated using logistic regression analyses.

**Table 5:** Questions (see **Appendix 1-4 in Supplementary Material**) and answers on attitudes and personal experience regarding genetic counseling and testing in general population (public) and experts.

Question					
		Approval: number (%)	Rejection: number (%)	Logistic regression	sign.
Attitudes					
Qu. 22/1: “How useful do you think pharmacogenetic tests are for mental disorders?” (Likert-scale)	Experts	116 (80)	29 (20)	F=0.639, df=2	p=0.530
Qu. 29: “Do you think genetic testing can help predict response to drug therapy for mental disorders or help choose a drug that is right for you?” (dichotomous)	Public	790 (79)	210 (21)	omnibus test chi-square 4.216, df=4	p=0.378
Personal Experience					
Qu. 7: “Have you already taken advantage of genetic counseling for mental disorder (because of your own conspicuous features or conspicuous features in members of the family)?” (dichotomous)	Public	80 (8)	920 (92)	omnibus test chi-square 83.432, df=4,	p<0.001*
Qu. 11: “Have you ever had genetic testing done?” (dichotomous)	Public	90 (9)	910 (91)	omnibus test chi-square 17.872, df=4	p=0.001**
Qu. 2: “Do you provide genetic counseling for mental disorders yourself or have you provided genetic counseling for mental disorders yourself?” (dichotomous)	Experts	57 (39)	88 (61)	omnibus test chi-square 5.200, df=2	p=0.074
Qu. 24: “Do you carry out or have you requested genetic tests for mental disorders to determine a diagnosis?” (dichotomous)	Experts	41 (28)	104 (72)	omnibus test chi-square 1.884, df=2	p=0.390

*A history of mental illness made it more likely to have received genetic counseling.

**Indiviuals who were younger, had lower levels of education and had a diagnosed mental disorder were more likely to have undergone genetic testing.

Influence of psycho-sociodemographic factors on the answering was calculated by logistic regression analyses.

### 2.4 Ethical standards

This project was submitted to and approved by the ethics committee of Danube University Krems, Austria. The committee determined that participation in the survey was voluntary, the participation process was anonymous, and therefore, written informed consent was not required. The study adhered to the ethical standards outlined in the 1964 Declaration of Helsinki and its subsequent amendments. It is important to note that the manuscript does not include clinical studies or patient data.

## 3 Results

### 3.1 Attitudes and knowledge in genetic counseling and diagnostic testing public and experts

Firstly, in both groups a majority considered genetic counseling in mental disorders as important (see **Table 3**). The public exhibited a stronger inclination towards considering genetic counseling for mental disorders as essential compared to experts. Specifically, 68.8% of the public expressed approval (Likert-scale mean 2.03), whereas 54.2% of experts did so (Likert-scale mean 2.37). This difference in response behavior was statistically significant (Pearson chi-square 12.183; df=1; p<0.001). Linear regression analyses (ANOVA) conducted to explore the response behavior in both groups did not reveal any significant influences from the independent variables (general population: F=0.910; df=4; p=0.457; experts: F=0.916; df=2; p=0.403). In public genetic counseling is said to be useful in the context of mental and even more in physical illnesses (Likert-scale mean 2.03 versus 1.80).

Secondly, when queried about the utility of genetic testing for diagnosing mental disorders, a significant contrast emerged between the public and experts (see **Table 3**). The public was more likely to view genetic testing as useful, with 67.9% offering affirmative responses (Likert-scale mean 2.11). In contrast, only 17.2% of experts shared this perspective (Likert-scale mean 3.34). This divergence was statistically significant (Pearson chi-square 137.236; df=1; p<0.001). Consequently, as in the previous case, linear regression analyses (ANOVA) was conducted to investigate response behavior in both groups to uncover significant influences from the independent variables (general population: F=3.081; df=4; p=0.016; experts: F=0.785; df=2; p=0.458). In the general population, age has a significant influence on the assessment of the usefulness of genetic testing for mental illnesses to determine the diagnosis. The older, the more useful these tests are perceived to be. However, the quality of the model is extremely low with an adjusted R-squared of 0.008. No influence on the answers by age and gender was seen in experts. Genetic testing is found to be useful by the public for determining a diagnosis for mental and even more in physical illness (Likert-scale mean 2.11 and 1.91). The anticipated positive consequences of genetic testing clearly outweigh the negative ones, 62% of the public see genetic testing as positive for psychiatric disorders. The public considers it positive to gain certainty by diagnostic genetic testing (64%) and to find inner peace through the test (63%). Most people (60%) would find the information that their own mental illness is genetically determined to be reassuring, in people with diagnosed mental illness even 65%. 15% believe that genetic testing has negative consequences in connection with mental illnesses. For those with higher education, the proportion is 20%. The negative consequences they see primarily in following aspects: high level of fear about the test result (59%), concern about stigmatization (48%), feelings of guilt about possible inheritance (38%), problems with insurance (24%), employers (22%), family (16%) and friends (11%). A not insignificant proportion of experts (overall 41%) believe in stigmatization and discrimination against mentally ill people as a result of genetic testing, specifically 49% in the age group above 55 years, 67% if rejecting the meaningfulness of even genetic counseling and only 29% if having completed a special training by Austrian Medical Association, the diploma in genetics.

Lastly, the question of whether genetic tests for diagnostic purposes were currently available was posed, and the response behavior was once again compared between the public and experts (see **Table 3**). A smaller proportion of the public (26.2%) believed that genetic tests with the capability to make psychiatric diagnoses were currently available, in contrast to experts, among whom 35.8% held this belief. Although the response behavior differed, it did so with a relatively high margin of error (Pearson chi-square 5.447; df=1; p=0.02). Experts, who think diagnostic genetic tests are available, think there are genetic tests for organic psychiatric disorders (78%), for schizophrenia (45%), for affective disorders (37%), for developmental disorders (35%) and for intellectual disability (33%). Again, logistic regression analyses conducted to explore response behavior did not reveal significant influences from the independent variables (general population: omnibus test chi-square 2.788; df=4; p=0.594; experts: omnibus test chi-square 3.420; df=2; p=0.181).

### 3.2 Knowledge in genetic counseling in experts

A minority (28%) reported having received specific training and education in genetic counseling. A notable correlation was observed with logistic regression between age and training in this domain. Specifically, as the age of the interviewed experts increased, they were more likely to have received training (omnibus test chi-square 18.033; df=2; p<0.001). This suggests that older experts in the field were more inclined to have formal training in genetic counseling (see **Table 4**).

### 3.3 Interest in genetic counseling and testing in public

A significant majority (79%) expressed their willingness to undergo genetic counseling if they perceived a need, because they themselves or relatives have a diagnosis of a mental illness (see **Table 4**). However, logistic regression analysis did not reveal any associations with independent variables, indicating that this desire was consistent across different demographic groups (omnibus test chi square 2.355; df=4; p=0.671). Similarly, when asked whether they would opt for genetic testing if it were offered to them, 74% responded affirmatively. Notably, the likelihood of agreeing to genetic testing increased among individuals who had already received a diagnosis (omnibus test chi-square 15.081; df=4; p=0.005). It’s important to acknowledge that this result carries a relatively high margin of error. Regarding the desire for pharmacogenetic testing in the event of non-response to drug therapy, the public showed no clear preference, with 51% in favor and 49% against. Linear regression analysis testing the independent variables did not identify any significant influences on this response (F=0.117; df=4; p=0.976).

### 3.4 Attitudes in pharmacogenetic testing in public and experts

Both groups share a strong consensus regarding the significance and utility of pharmacogenetic testing for mental disorders, with 79% (public) and 80% (experts) approval rates (Likert-scale mean in experts 1.76). It’s noteworthy that when analyzing the response behavior in both groups through logistic or linear regression analyses, the independent variables did not exhibit significant influences (general population: omnibus test chi-square 4.216; df=4; p=0.378; experts: F=0.639; df=2; p=0.530). This underscores the substantial agreement across these two cohorts on the importance and helpfulness of pharmacogenetic testing. 63% of experts consider pharmacokinetic testing to be very useful (Likert-scale mean 1.56), and 47% consider pharmacodynamic testing to be very useful (Likert-scale mean 1.93). Three quarters (75%) of the public see the positive consequences of a tailored treatment approach (see **Table 5**).

### 3.5 Personal experience in genetic counseling and testing in public and experts

In the public, 8% reported having undergone genetic counseling for mental disorders, with 60% of these cases related to depressive disorders and 18% to anxiety disorders. Logistic regression analysis revealed a significant association: individuals with a history of mental illness were more likely to have received genetic counseling (omnibus test chi-square 83.432; df=4; p<0.001). Similarly, 9% of the public had undergone genetic testing, 43% of these because of a physical illness, 41% in the context of pharmacogenetics and 33% because of a psychiatric illness (see **Table 5**). Logistic regression analysis showed that individuals who were younger, had lower levels of education, and had a diagnosed mental disorder were more likely to have undergone genetic testing (omnibus test chi-square 17.872; df=4; p=0.001).

Among experts, a minority had experience in genetic counseling for mental illnesses (39%). Logistic regression analyses did not identify significant influences of age and gender on response behavior in this expert group (omnibus test chi square 5.200; df=2; p=0.074). Genetic counseling was mainly provided for diseases from the schizophrenia spectrum (71%), followed by affective disorders (64%) and organic psychiatric disorders (54%). The clients in genetic counseling were mainly patients themselves (93%) and women (93%). The advice was mostly based on family history (91%) and, to a lesser extent, on genetic tests (50%). Experts are only moderately satisfied with training in genetic counseling in Austria. A majority (63%) is clearly critical of this, only 9% consider it to be satisfactory (Likert-scale mean 3.86). 28% of experts had experience with diagnostic genetic testing, if done, especially provided for organic psychiatric disorders (67%), schizophrenia (43%) and affective disorders (38%). Logistic regression analyses did again not identify significant influences of age and gender on response behavior in this expert group (omnibus test chi-square 1.884; df=2; p=0.390) (see **Table 5**).

## 4 Discussion

Genetic counseling and testing in psychiatry warrant attention, but little reliable knowledge is available about attitude, knowledge, personal experience and interest. There is one study reporting on opinions, knowledge and practices in a sample of professionals in Europe who are interested in the topic (46). But methods and questions used did not correspond to our work presented here, e.g. no sample of the public was surveyed and therefore no comparison is made with public samples. The authors highlight the lack of guidelines, knowledge, training and education in the field. This publication was created within a funded action of European Union, our group was participant and member of the action too. Our study aimed to investigate these gaps through a cross-sectional survey conducted in Austria, involving a sample of the adult web-active population and experts. This is the inaugural exploratory survey study on this subject in Austria.

### 4.1 Genetic counseling

Notably, in our study, genetic counseling was deemed important by both the public and experts, albeit slightly more so by the former. In contrast to the expressed interest and perceived importance of genetic counseling and testing in mental illness, our surveys revealed that very few individuals had taken advantage of genetic counseling (8%), especially those with psychiatric disorders (depressive and anxiety disorders). But only a quarter of our public sample had a mental illness, and genetic counseling is not offered to everyone with a psychiatric diagnosis. Experts also reported limited experience with genetic counseling (39%). To our knowledge, there are no available publications with surveys on public opinion regarding genetic counseling in mental illness. Few survey studies have been conducted with experts on genetic counseling in the context of mental disorders in other countries. In India, researchers surveyed 150 psychiatrists regarding their attitudes toward genetic counseling. A majority (92%) reported that patients often request it, but only 39% provided information on genetic aspects of the illnesses. Interestingly, experts in India rated the importance of genetic counseling higher (93%) than experts in Austria. However, psychiatrists in India expressed concerns about the adequacy of their knowledge, with 81% feeling insufficiently informed about the genetic aspects of mental illnesses (47). A study in Spain questioned experts (n=152) about their views on the genetics of psychiatric disorders and genetic counseling. The majority (59%) considered the establishment of genetic counseling institutions to be valuable, similar to Austria. Almost two-thirds (61%) strongly believed that psychiatric disorders have a genetic basis, but only 12% of patients inquired about genetic causes. Users (patients and relatives, n=959) were less convinced (47%) about a genetic basis, but 80% considered genetic counseling units important, although only 14% reported that their psychiatrists had discussed the issue (48). In the USA, a study involving genetic counselors (n=175) revealed a similarly high level of approval for genetic counseling, akin to India. However, only a minority (44%) offered it for mental disorders, despite 94% finding it important. Notably, genetic counselors believed that the value of counseling for mental illnesses was limited due to a lack of genetic data (49). In Australia and New Zealand, a survey of genetic counselors (n=44) explored their practices regarding mental disorders. The majority (75%) had never been involved with psychiatric issues, 72% did not feel confident in providing risk assessments for mental illnesses, and 95% expressed interest in specialist training (50).

Indeed, there are several studies on genetic counseling and testing among patients with specific mental illnesses and their relatives, although we cannot directly compare these findings with Austrian data. Here are some key findings from these studies: In a study conducted in the USA on genetic counseling in patients with schizophrenia (n=68) and their relatives (n=145), it was found that 0% of affected individuals and only 5% of relatives had received genetic counseling. Only a minority (6%) of respondents had been offered genetic counseling. However, a significant proportion (74% of relatives and 72% of patients) believed that counseling would be useful to them (51).

In summary, it is noteworthy that in Austria, experts assessed the value of genetic counseling as high, but as relatively lower compared to other countries, despite the fact that experts in these other studies seldom utilized it.

### 4.2 Diagnostic genetic testing

Regarding genetic testing, only 9% of the public stated that they had undergone genetic testing, mostly for physical illnesses (43%), followed by pharmacogenetics (41%) and mental disorders (33%). Notably, individuals with lower education levels and those with mental illness reported more frequent genetic testing. A minority of experts (28%) had experience with diagnostic genetic tests in mental illness. These findings are consistent with other surveys on similar topics. The divergence in public and expert opinion regarding diagnostic genetic testing, as observed in Austria, is an intriguing phenomenon. While the public believes in the utility of genetic tests for diagnosis, experts hold a contrasting view. This discrepancy raises questions as to whether expert consensus is adequately communicated to the public. It also underscores the importance of education campaigns and the need for experts to engage in dialogue with the public to align perspectives. Notably, we couldn’t find publications on this specific topic in other countries. Regarding the existence of such tests today, only a minority of both the public and experts believe they currently exist.

The following studies provide valuable insights into the attitudes and perceptions surrounding genetic testing (and counseling) for mental disorders in various populations and regions. In Denmark, a study involving psychiatric patients (n=397), their relatives (n=164), and students (n=100) explored attitudes and intentions regarding psychiatric genetic testing. A majority of respondents (66.6%) agreed that anyone who desires it should have the opportunity for testing, and 38% of patients expressed a desire for psychiatric genetic testing, regardless of treatment options (52). A comparative study between Cuba (n=720) and Denmark (n=491) examined attitudes toward genetic testing among students, patients with depression, and relatives of patients. Significant differences were observed between the two countries in terms of attitudes, knowledge, and opportunities for genetic testing. Notably, respondents in Cuba expressed more discomfort about psychiatric genetic research compared to those in Denmark. Additionally, more patients from Cuba (52% versus 26%) expressed fear about their ability to cope emotionally with the results of a genetic test (53). A study in the USA focused on patients with eating disorders (n=107) and their perspectives on genetic risk, testing, and counseling. The participants tended to overestimate the risk of their children developing an eating disorder and expressed interest in genetic counseling (61%) and testing (47%) related to their eating disorders (54). Surveys of the general population have also explored interest in genetic testing for specific mental illnesses or among those affected by these illnesses. For example, scientists conducted a survey in Australia (n=1,046) and found a high level of interest (60-63%) in depression-risk genotyping through a doctor, with interest positively associated with a personal history of mental illness, self-perceived high risk for depression, being female, and having no post-school education (55). The authors anticipated a strong demand for predictive genetic testing. Researchers in Australia surveyed genetic research participants in Australia (n=3,646) and members of the public in the UK and the USA (n=960) regarding their interest in genetic testing for mental illness (56). Preliminary analysis showed higher interest in learning about genetic predisposition for alcohol dependence, schizophrenia, and depression in the US sample compared to Australia and the UK. In the UK, only about a third of the public expressed a desire to know their genetic predisposition, while in the US, this figure rose to two-thirds, with the Australian sample falling in between the figures. In Sweden, a survey was conducted among parents of autistic children (n=868) and autistic adolescents and adults (n=213) to assess access, utilization, and awareness of genetic counseling and testing. The majority of respondents (65.8% of parents, 50% of patients) accepted genetic testing if referrals were done. However, only a minority (9.1% of parents, 2.8% of patients) had been informed about such offerings. Interestingly, a significant portion of respondents mistakenly believed that a genetic test was available to diagnose autism spectrum disorder (ASD) (16.2% of parents, 19.6% of patients) (57). In Canada, a survey of adults with autism (n=461) addressed the question of genetic testing. A significant proportion (74%) believed that genetic testing should only be conducted if the individual concerned can provide consent, and approximately half (49%) expressed the opinion that no genetic testing should be conducted at all. Only a minority of respondents felt that genetic testing should be routinely offered (35% in adults, 26% in children) (58).

### 4.3 Pharmacogenetic testing

Pharmacogenetic testing was considered important by both groups in our survey, aligning with similar studies that show overwhelming approval of pharmacogenetic testing among professionals. In an US survey of psychiatric patients (n=598), the focus was on pharmacogenetic testing. Respondents expressed interest in this type of testing, with an average rating of 4.16 on a Likert-scale (from 1 “strongly disagree” to 7 “strongly agree”). However, they reported having limited information on the topic (average rating of 2.09) and a preference for seeking information to reduce uncertainty (average rating of 5.34) (59). Scientists conducted a study surveying US neurologists and psychiatrists, finding that a significant proportion of neurologists had ordered genetic testing in the past six months, albeit a small fraction for pharmacogenetics (60). Among psychiatrists, a minority had referred for testing, particularly for pharmacogenetics. Overall, there was a consensus that genetic testing should be used more frequently, with many respondents indicating a need for further education in this field. Concerns were raised about the potential psychological harm to patients, and calls for better legislation to protect patients’ genetic data. Similarly, a survey of US psychiatrists (61) revealed that 94.6% believed pharmacogenetic testing would be useful for making pharmacological decisions, and 85.1% anticipated these tests becoming standard practice in psychiatry. A substantial portion (72.6%) believed that including genetic counselors in psychiatric care would be beneficial. In Singapore, a survey among 194 experts, including doctors and pharmacists, found that 80.9% considered psychiatric pharmacogenomic testing useful for identifying suitable treatments, particularly in patients with drug intolerance (62). However, only 46.4% felt competent to order such tests. Scientists in the USA published a study on attitudes and practices regarding diagnostic testing and pharmacogenetics among child and adolescent psychiatrists (n=958) (63). Most respondents reported having used genetic testing in the past year, with pharmacogenetic tests being the most frequently ordered (32%). However, 45% rated their knowledge of genetic testing practice guidelines as poor. Nonetheless, 73% perceived pharmacogenetic testing as at least slightly useful in child and adolescent psychiatry.

### 4.4 Strength and limitations

The strength of our study is that it is the first of its kind in Austria. Another strength is the sample size in both groups, which allows to draw reasonable conclusions. A limitation is that the selection of the two groups surveyed did not focused on the groups most central to implementation in practice, namely psychiatric patients and psychiatrists. In our sample of the public we had 24.5% individuals with psychiatric diagnosis and in the expert sample we had 83.4% psychiatrists. Another limitation is that it was not assessed what genetic counseling actually included for participants who reported experience with genetic counseling. Was it a conversation around family history, was it done in more than one session, and was information given about genetic testing? Additionally, we do not have details on the counseling provided by experts.

## 5 Conclusion

These results collectively illustrate the complexity of attitudes and behaviors related to genetic counseling and testing for mental disorders, underscoring the need for further research and education in this field to bridge the gap between interest and utilization. Our study represents a pioneering effort in Austria, and as far as our research extends, there is no analogous study worldwide that employs comparable methods and inquiries: namely the survey of a representative public sample and the direct comparison of the response behavior with a group of experts regardless to the level, quality and source of prior knowledge in the topic in both samples. Nevertheless, a comprehensive review of literature and research in this domain reveals a noteworthy convergence of outcomes. Specifically, it underscores the substantial interest expressed by the public and their growing demand for genetic counseling. Moreover, it emphasizes the increasing significance of genetic testing, particularly in the realm of pharmacogenetics. This ascendancy in importance is already evident, particularly in cases involving treatment-resistant diseases or a high incidence of adverse drug reactions.

Drawing from these valuable insights, we strongly assert that genetic counseling and a broader understanding of the genetic underpinnings of mental illnesses must be given a more prominent role in education and training of medical professionals. Genetic counseling not only revolves around what can be tested, but also includes education about the limitations of the current knowledge, e.g. that there is currently no valid diagnostic genetic testing possible for most psychiatric disorders. These facets are essential components of comprehensive patient care, and their inclusion in medical training programs stands to enhance healthcare delivery.

## Data Availability

The raw data supporting the conclusions of this article will be made available by the authors, without undue reservation.
