# Supplementary material for: A survey in Austria supports the significance of genetic counseling and pharmacogenetic testing for mental illness
Source: Front Psychiatry. 2024 Oct 3;15:1436875. doi: 10.3389/fpsyt.2024.1436875 (PMC11484073; doi:10.3389/fpsyt.2024.1436875)
Supplement: Supplementary file 1 [file DataSheet1.zip › Appendix 1.DOCX]

Appendix 1

Questionnaire for the general population (in German) [Akimova & Shahriar Izadi, 2021]:

Genetische Beratung und Testung aus Sicht der österreichischen Bevölkerung

In der heutigen Umfrage widmen wir uns einem „medizinischen Thema“.

Dabei geht es nicht darum, dass Sie bestimmte Dinge konkret wissen, sondern um Ihre persönliche gefühlsmäßige Einschätzung.

1. Wurde bei Ihnen jemals eine psychische Krankheit diagnostiziert?

1 ja

2 nein

1. Wenn ja lt. Frage 1: Welche? (Mehrfachnennungen möglich)

- Demenz
- Problematischer Alkoholkonsum
- Problematischer Konsum von Substanzen (außer Alkohol)
- Schizophrenie oder Psychose
- Depression
- Bipolare Störung
- Angststörung
- Posttraumatische Belastungsstörung
- Essstörung
- Persönlichkeitsstörung
- Andere

1. Wie sinnvoll halten Sie genetische Beratung* bei psychischen Krankheiten?

*Unter genetischer Beratung versteht man ein ärztliches Gespräch, das PatientInnen über eventuell vorhandene genetische Störungen und daraus resultierende Krankheiten oder Risiken aufklärt.

Bitte benützen Sie für Ihr Urteil eine Skala von 1 - 5, wobei

1 bedeutet: sehr sinnvoll

5 bedeutet: gar nicht sinnvoll

Dazwischen können Sie abstufen!

|  | Sehr sinnvoll | 1 | 2 | 3 | 4 | 5 | Gar nicht sinnvoll |
| --- | --- | --- | --- | --- | --- | --- | --- |

1. Wie sinnvoll halten Sie genetische Beratung bei körperlichen Krankheiten?

Bitte benützen Sie für Ihr Urteil eine Skala von 1 - 5, wobei

1 bedeutet: sehr sinnvoll

5 bedeutet: gar nicht sinnvoll

Dazwischen können Sie abstufen!

|  | Sehr sinnvoll | 1 | 2 | 3 | 4 | 5 | Gar nicht sinnvoll |
| --- | --- | --- | --- | --- | --- | --- | --- |

1. Würden Sie sich bei Bedarf (wegen psychischer Krankheiten) genetisch beraten lassen?

1 ja

2 nein

1. Wenn ja lt. Frage 5: In welchem Zusammenhang? (Mehrfachnennungen möglich)

- Bei eigener psychischer Krankheit
- Bei psychischer Krankheit von Familienangehörigen
- Bei Kinderwunsch
- Bei Partnerwahl bzw. Partnerinwahl
- Anderes

1. Haben Sie selbst schon genetische Beratung bei psychischer Krankheit in Anspruch genommen (wegen eigener Auffälligkeiten oder Auffälligkeiten innerhalb der Familie)?

1 ja

2 nein

1. Wenn ja lt. Frage 7: Bei welcher psychischen Krankheit oder Problemen? (Mehrfachnennungen möglich) (bei Ihnen selbst oder im familiären Umfeld)

- Demenz
- Problematischer Alkoholkonsum
- Problematischer Konsum von Substanzen (außer Alkohol)
- Schizophrenie oder Psychose
- Depression
- Bipolare Störung
- Angststörung
- Posttraumatische Belastungsstörung
- Essstörung
- Persönlichkeitsstörung
- Suizide in der Familie
- Andere

1. Wen würden Sie aufsuchen, wenn Sie genetisch beraten werden wollen?

- Hausärztin oder Hausarzt
- Fachärztin oder Facharzt je nach Diagnose
- Krankenhaus
- Institut für Humangenetik
- Andere

1. Sollte genetische Beratung in Österreich durch eine Berufsgruppe mit eigener, spezieller Ausbildung angeboten werden?

- Ja, durch eigene Berufsgruppe Genetische Berater*in
- Nein, soll durch Ärztin oder Arzt gemacht werden

1. Wurde bei Ihnen jemals eine genetische Testung** durchgeführt?

**Unter genetischer Testung versteht man genetische Untersuchungen (z.B. DNA-Analysen) zur Bestimmung oder Verbesserung der Diagnose oder Therapie.

1 ja

2 nein

1. Wenn ja lt. Frage 11: In welchem Zusammenhang? (Mehrfachnennungen möglich)

- Bei psychischer Krankheit
- Bei körperlicher Krankheit
- Um bei Krankheit die für mich passende Therapie auszuwählen/den „richtigen“ Therapieansatz zu bestimmen
- Anderes

1. Wenn man Ihnen vorschlagen würde, sich genetisch testen zu lassen, würden Sie es machen?

1 ja

2 nein

1. Wenn nein lt. Frage 13: Warum nicht? (Mehrfachnennungen möglich)

- Aus Angst vor dem Ergebnis der Testung
- Aus Sorge, stigmatisiert zu werden, wenn ich jemandem davon erzähle (dass ich den Test gemacht habe bzw. eine Krankheit habe)
- Weil ich bei Feststellen einer Krankheit Schuldgefühle hätte, dass ich diese Krankheit vererben könnte
- Weil ich Probleme mit der Versicherung erwarte
- Weil ich Probleme mit der/m Arbeitgeber*in erwarte
- Weil ich Probleme mit der Familie erwarte
- Weil ich Probleme mit Freunden erwarte
- Weil ich keine Notwendigkeit sehe
- Anderes

1. Glauben Sie, dass genetisches Testen bei psychischen Krankheiten negative Folgen hat?

1 ja

2 nein

1. Wenn ja lt. Frage 15: In welcher Hinsicht? (Mehrfachnennungen möglich)

- Deutlicher Anstieg der Angst vor dem Ergebnis der Testung
- Erhöhte Sorge, stigmatisiert zu werden, wenn ich jemandem davon erzähle (dass ich den Test gemacht habe bzw. eine Krankheit habe bzw. ein Risiko für eine Krankheit habe)
- Hohes Maß an Schuldgefühlen bei Feststellen einer Krankheit, dass ich diese Krankheit vererben könnte
- Probleme mit der Versicherung
- Probleme mit der/m Arbeitgeber*in
- Probleme mit der Familie
- Probleme mit Freunden
- Andere

1. Glauben Sie, dass genetisches Testen bei psychischen Krankheiten positive Auswirkungen hat?

1 ja

2 nein

1. Wenn ja lt. Frage 17: In welcher Hinsicht? (Mehrfachnennungen möglich)

- Die Sicherheit zu bekommen, dass ich die Krankheit habe oder auch nicht habe/Gewissheit erlangen
- Innerliche Beruhigung, dass ich Bescheid weiß
- Im Krankheitsfall Erhalt einer besseren Behandlung, d.h. zielgerichtete, spezifische Behandlung, die auf mein Krankheitsbild abgestimmt ist
- Anderes

1. Würden Sie das Ergebnis einer genetischen Testung anderen Menschen mitteilen?

1 ja

2 nein

1. Wenn ja lt. Frage 19: Wem? (Mehrfachnennungen möglich)

- Eltern
- Partner*in
- Kindern
- Geschwistern
- Freunden
- Arbeitgeber*in
- Anderen

1. Sollen genetische Testungen bei psychischen Krankheiten von der Sozialversicherung bezahlt werden?

1 ja

2 nein

1. Würden Sie auch privat für genetische Testungen bei psychischen Krankheiten bezahlen, d.h. die Kosten dafür selbst übernehmen?

1 ja

2 nein

1. Stehen Ihrer Meinung nach, derzeit genetische Testungen zur Verfügung, die die Diagnosestellung psychischer Krankheiten ermöglichen?

1 ja

2 nein

1. Wenn ja lt. Frage 23: Erlauben die derzeit verfügbaren genetischen Testungen eine Einschätzung des Risikos für einen Menschen, eine bestimmte psychische Krankheit zu bekommen?

1 ja

2 nein

1. Wie sinnvoll halten Sie genetische Testungen bei psychischen Krankheiten zur Bestimmung einer Diagnose?

Bitte benützen Sie für Ihr Urteil eine Skala von 1 - 5, wobei

1 bedeutet: sehr sinnvoll

5 bedeutet: gar nicht sinnvoll

Dazwischen können Sie abstufen!

|  | Sehr sinnvoll | 1 | 2 | 3 | 4 | 5 | Gar nicht sinnvoll |
| --- | --- | --- | --- | --- | --- | --- | --- |

1. Wie sinnvoll halten Sie genetische Testungen bei körperlichen Krankheiten zur Bestimmung einer Diagnose?

Bitte benützen Sie für Ihr Urteil eine Skala von 1 - 5, wobei

1 bedeutet: sehr sinnvoll

5 bedeutet: gar nicht sinnvoll

Dazwischen können Sie abstufen!

|  | Sehr sinnvoll | 1 | 2 | 3 | 4 | 5 | Gar nicht sinnvoll |
| --- | --- | --- | --- | --- | --- | --- | --- |

1. Würde es Sie beruhigen, wenn Sie erfahren sollten, dass genetische Ursachen für Ihre psychische Störung verantwortlich sind?

1 ja

2 nein

1. Glauben Sie, dass die Entdeckung genetischer Ursachen bei psychischen Krankheiten helfen könnte, eine bessere, spezifischere Behandlung vorzunehmen?

1 ja

2 nein

1. Glauben Sie, dass genetische Testungen helfen können, das Ansprechen auf eine medikamentöse Therapie bei einer psychischen Krankheit vorauszusagen oder ein für Sie passendes Medikament auszuwählen?

1 ja

2 nein

1. Glauben Sie, dass genetische Testungen helfen können, das Ansprechen auf eine medikamentöse Therapie bei einer körperlichen Krankheit vorauszusagen oder ein für Sie passendes Medikament auszuwählen?

1 ja

2 nein

1. Wie wahrscheinlich wäre es, dass Sie sich genetisch testen lassen, wenn Sie starke Nebenwirkungen auf ein Medikament haben?

Bitte benützen Sie für Ihr Urteil eine Skala von 1 - 5, wobei

1 bedeutet: sehr wahrscheinlich

5 bedeutet: gar nicht wahrscheinlich

Dazwischen können Sie abstufen!

|  | Sehr wahrscheinlich | 1 | 2 | 3 | 4 | 5 | Gar nicht wahrscheinlich |
| --- | --- | --- | --- | --- | --- | --- | --- |

1. Wie wahrscheinlich wäre es, dass Sie sich genetisch testen lassen, wenn Sie auf eine medikamentöse Therapie NICHT ansprechen?

Bitte benützen Sie für Ihr Urteil eine Skala von 1 - 5, wobei

1 bedeutet: sehr wahrscheinlich

5 bedeutet: gar nicht wahrscheinlich

Dazwischen können Sie abstufen!

|  | Sehr wahrscheinlich | 1 | 2 | 3 | 4 | 5 | Gar nicht wahrscheinlich |
| --- | --- | --- | --- | --- | --- | --- | --- |

1. Haben Sie jemals eine genetische Testung (unabhängig für welchen Zweck) im Internet angefordert?

1 ja

2 nein

1. Wenn ja lt. Frage 33: Weswegen?

- Um das Risiko für Krankheiten zu bestimmen
- Um etwas über meine Abstammung/Herkunft zu erfahren
- Anderes

1. Wenn ja lt. Frage 33: Haben Sie eine solche (über Internet angeforderte) genetische Testung mit einer Ärztin oder einem Arzt nachbesprochen?

1 ja

2 nein

Abschließend noch ein paar Angaben zu Ihrer Person:

A. Geschlecht:

1 männlich

2 weiblich

B. Alter: ________ Jahre (genau eintragen!)

1 18 - 30 Jahre

2 31 - 50 Jahre

3 über 50 Jahre

C. Bundesland:

1 Wien 4 Steiermark 7 Salzburg

2 Niederösterreich 5 Kärnten 8 Tirol

3 Burgenland 6 Oberösterreich 9 Vorarlberg

D. Wohnortgröße:

1 Bis 2.000 Einwohner 4 Bis 50.000 Einwohner

2 Bis 5.000 Einwohner 5 Über 50.000 Einwohner

3 Bis 20.000 Einwohner 6 Wien

E. Beruf: In welche dieser Berufsgruppen würden Sie sich selbst einstufen?

1 freie Berufe, Selbständige, leitende Angestellte und Beamte

2 nicht-leitende Angestellte und Beamte

3 Arbeiter*in

4 Landwirt*in

5 in Ausbildung / Schüler*in / Student*in

6 nicht berufstätig (im Haushalt tätig / in Karenz / arbeitssuchend)

7 Pensionist*in

F. Haushaltsnettoeinkommen monatlich:

1. bis 1.000 Euro
2. bis 1.500 Euro
3. bis 2.000 Euro
4. bis 2.500 Euro
5. bis 3.000 Euro
6. bis 3.500 Euro
7. bis 4.000 Euro
8. über 4.000 Euro

G. Schulbildung: Welche ist Ihre höchste abgeschlossene Schulbildung?

1 Kein Abschluss, Pflichtschule

2 Berufs-, Fachschule, mittlere Lehranstalt

3 AHS, BHS, Mittelschule ohne Matura

4 Matura

5 Fachhochschule, Universität
