# Supplementary material for: A survey in Austria supports the significance of genetic counseling and pharmacogenetic testing for mental illness
Source: Front Psychiatry. 2024 Oct 3;15:1436875. doi: 10.3389/fpsyt.2024.1436875 (PMC11484073; doi:10.3389/fpsyt.2024.1436875)
Supplement: Supplementary file 1 [file DataSheet1.zip › Appendix 2.DOCX]

Appendix 2

Questionnaire for experts (in German) [Akimova & Shariar Izadi, 2021]

Genetische Beratung und Testung aus Expert*innensicht

Guten Tag! Mein Name ist ………...vom Österreichischen Gallup Institut.

Wir führen zurzeit eine Studie im medizinischen Bereich durch und befragen FachärztInnen bzw. ExpertInnen zur Situation der genetischen Beratung und Testung in Österreich.

Die Ergebnisse dieser Studie sollen publiziert und bei wissenschaftlichen Kongressen vorgestellt werden.

Die Befragung wird ca. 10 Minuten Zeit in Anspruch nehmen.

Ihre Angaben werden selbstverständlich absolut vertraulich behandelt und anonym ausgewertet.

Darf ich Ihnen dazu ein paar Fragen stellen?

Wenn ja, weiter mit Interview (entweder sofort oder Terminvereinbarung).

Wenn nein, bedanken und ENDE.

1. Wie sinnvoll halten Sie genetische Beratung bei psychischen Störungen?

1 bedeutet: sehr sinnvoll

5 bedeutet: gar nicht sinnvoll

Dazwischen können Sie abstufen!

|  | Sehr sinnvoll | 1 | 2 | 3 | 4 | 5 | Gar nicht sinnvoll |
| --- | --- | --- | --- | --- | --- | --- | --- |

1. Führen Sie selbst genetische Beratung bei psychischen Störungen durch oder haben Sie selbst genetische Beratung bei psychischen Störungen durchgeführt?

1 ja

2 nein

1. Wenn ja lt. Frage 2: Wie oft haben Sie in den letzten 10 Jahren genetische Beratung bei psychischen Störungen durchgeführt?

- 1-10 mal
- 11-20 mal
- Mehr als 20 mal

1. Wenn ja lt. Frage 2: Wen haben Sie bezüglich psychischer Störungen genetisch beraten? (Mehrfachnennungen möglich)

1 PatientInnen

2 Verwandte

3 Andere, und zwar:_______________________

1. Wenn ja lt. Frage 2: Bei welchen Zielgruppen haben Sie diese Beratungen durchgeführt? (Mehrfachnennungen möglich)

1 Frauen

2 Männer

3 Kinder und Jugendliche

1. Wurden Sie von Patient*innen um genetische Beratung gebeten, nachdem diese genetische Testungen im Internet bestellt hatten und das Ergebnis ohne Beratung direkt, unmittelbar vom Labor mitgeteilt bekommen hatten („Direct-to-consumer-Test“, DTC; unter Ausschaltung der Fachleute)?

1 ja – wie oft ist das bereits vorgekommen? _____________(Anzahl einfügen, nur 1 Zahl)

2 nein

1. Wenn ja lt. Frage 2: Für welche psychischen Störungen haben Sie genetische Beratung gemacht? (Mehrfachnennungen möglich)

- ICD-10 F0: u.a. Organische Störungen
- ICD-10 F1: u.a. Psychische Störungen durch psychotrope Substanzen
- ICD-10 F2: u.a. Schizophrenien, schizotype und wahnhafte Störungen
- ICD-10 F3: u.a. Affektive Störungen
- ICD-10 F4: u.a. Neurotische und Somatoforme Störungen
- ICD-10 F5: u.a. Verhaltensauffälligkeiten mit körperlichen Störungen und Faktoren
- ICD-10 F6: u.a. Persönlichkeitsstörungen
- ICD-10 F7: u.a. Intelligenzminderung
- ICD-10 F8: u.a. Entwicklungsstörungen
- ICD-10 F9: u.a. Verhaltens- und emotionale Störungen mit Beginn in der Kindheit und Jugend

1. Wenn ja lt. Frage 2: Beraten Sie anhand von…. (Mehrfachnennungen möglich)

1 Familienanamnese

2 Ergebnissen von genetischen Tests

3 Anderes und zwar:____________________________

1. Wenn ja lt. Frage 2: Haben Sie vor oder nach genetischer Beratung PatientInnen zu genetischen Testungen hinsichtlich psychischer Störungen zugewiesen?

1 ja

2 nein

1. Haben Sie vor oder nach genetischer Beratung PatientInnen zu spezialisierten Einrichtungen, wie z.B. Institute für Humangenetik, zugewiesen?

1 ja

2 nein

ALLE:

1. Wie zufriedenstellend erachten Sie die Ausbildung zu genetischer Beratung in Österreich?

1 bedeutet: sehr zufriedenstellend

5 bedeutet: gar nicht zufriedenstellend

Dazwischen können Sie abstufen!

|  | Sehr zufriedenstellend | 1 | 2 | 3 | 4 | 5 | Gar nicht zufriedenstellend |
| --- | --- | --- | --- | --- | --- | --- | --- |

1. Haben Sie sich speziell in genetischer Beratung fort- bzw. weitergebildet (im In- oder Ausland)?

1 ja – wenn ja, auf welche Art und Weise? ________________________

2 nein

1. Sollte genetische Beratung in Österreich durch eine Berufsgruppe mit eigener, spezieller Ausbildung angeboten werden?

- Ja, durch eigene Berufsgruppe „Genetische Berater*in“
- Nein, soll durch Ärztin/Arzt gemacht werden

1. Stehen derzeit genetische Testungen zur Verfügung, deren Einsatz die Diagnosestellung psychischer Störungen ermöglichen?

1 ja

2 nein

1. Wenn ja lt. Frage 14: Bei welchen Indikationen?

- ICD-10 F0: u.a. Organische Störungen
- ICD-10 F1: u.a. Psychische Störungen durch psychotrope Substanzen
- ICD-10 F2: u.a. Schizophrenien, schizotype und wahnhafte Störungen
- ICD-10 F3: u.a. Affektive Störungen
- ICD-10 F4: u.a. Neurotische und Somatoforme Störungen
- ICD-10 F5: u.a. Verhaltensauffälligkeiten mit körperlichen Störungen und Faktoren
- ICD-10 F6: u.a. Persönlichkeitsstörungen
- ICD-10 F7: u.a. Intelligenzminderung
- ICD-10 F8: u.a. Entwicklungsstörungen
- ICD-10 F9: u.a. Verhaltens- und emotionale Störungen mit Beginn in der Kindheit und Jugend

1. Stehen derzeit genetische Testungen zur Verfügung, deren Einsatz eine Verlaufs-Prognose einer psychischen Störung zulassen?

1 ja

2 nein

1. Wenn ja lt. Frage 16: Bei welchen Indikationen?

- ICD-10 F0: u.a. Organische Störungen
- ICD-10 F1: u.a. Psychische Störungen durch psychotrope Substanzen
- ICD-10 F2: u.a. Schizophrenien, schizotype und wahnhafte Störungen
- ICD-10 F3: u.a. Affektive Störungen
- ICD-10 F4: u.a. Neurotische und Somatoforme Störungen
- ICD-10 F5: u.a. Verhaltensauffälligkeiten mit körperlichen Störungen und Faktoren
- ICD-10 F6: u.a. Persönlichkeitsstörungen
- ICD-10 F7: u.a. Intelligenzminderung
- ICD-10 F8: u.a. Entwicklungsstörungen
- ICD-10 F9: u.a. Verhaltens- und emotionale Störungen mit Beginn in der Kindheit und Jugend

1. Erlauben die derzeit verfügbaren genetischen Testungen eine Einschätzung des Risikos für einen Menschen bestimmte psychische Störungen zu erleiden?

1 ja

2 nein

1. Wenn ja lt. Frage 18: Bei welchen Indikationen?

- ICD-10 F0: u.a. Organische Störungen
- ICD-10 F1: u.a. Psychische Störungen durch psychotrope Substanzen
- ICD-10 F2: u.a. Schizophrenien, schizotype und wahnhafte Störungen
- ICD-10 F3: u.a. Affektive Störungen
- ICD-10 F4: u.a. Neurotische und Somatoforme Störungen
- ICD-10 F5: u.a. Verhaltensauffälligkeiten mit körperlichen Störungen und Faktoren
- ICD-10 F6: u.a. Persönlichkeitsstörungen
- ICD-10 F7: u.a. Intelligenzminderung
- ICD-10 F8: u.a. Entwicklungsstörungen
- ICD-10 F9: u.a. Verhaltens- und emotionale Störungen mit Beginn in der Kindheit und Jugend

1. Wie sinnvoll erachten Sie genetische Testungen bei psychischen Störungen zur Bestimmung einer Diagnose?

1 bedeutet: sehr sinnvoll

5 bedeutet: gar nicht sinnvoll

Dazwischen können Sie abstufen!

|  | Sehr sinnvoll | 1 | 2 | 3 | 4 | 5 | Gar nicht sinnvoll |
| --- | --- | --- | --- | --- | --- | --- | --- |

1. Glauben Sie, dass genetische Testungen bei psychischen Störungen das Stigma und die Diskriminierung psychisch Kranker erhöhen?

1 ja

2 nein

1. Wie sinnvoll halten Sie die folgenden Testungen bei psychischen Störungen?

1 bedeutet: sehr sinnvoll

5 bedeutet: gar nicht sinnvoll

Dazwischen können Sie abstufen!

|  |  | **sehr**  **sinnvoll** |  |  | |  | **gar nicht**  **sinnvoll** |
| --- | --- | --- | --- | --- | --- | --- | --- |
|  | Pharmako**genetische** Testungen (untersuchen den Einfluss der genetischen Ausstattungen auf Arzneimittelwirkungen) | 1 | 2 | | 3 | 4 | 5 |
|  | Pharmako**kinetische** genetische Testungen (Pharmakokinetik betrifft den Metabolismus der Medikamente, z.B. durch die Familie der CYP450 Enzyme. Es können die Gene dieser Enzyme untersucht werden) | 1 | 2 | | 3 | 4 | 5 |
|  | Pharmako**dynamische** genetische Testungen (untersuchen den Einfluss der genetischen Ausstattungen der Zielstrukturen der Medikamente, z.B. Neurotransmitter-Rezeptor-Gene, auf Arzneimittelwirkungen) | 1 | 2 | | 3 | 4 | 5 |

1. Haben Sie ein eigenes Labor für genetische Testungen?

1 ja

2 nein

1. Führen Sie selbst genetische Testungen bei psychischen Störungen zur Bestimmung einer Diagnose durch oder haben diese angefordert?

1 ja

2 nein

1. Wenn ja lt. Frage 24: Bei welchen Indikationen?

- ICD-10 F0: u.a. Organische Störungen
- ICD-10 F1: u.a. Psychische Störungen durch psychotrope Substanzen
- ICD-10 F2: u.a. Schizophrenien, schizotype und wahnhafte Störungen
- ICD-10 F3: u.a. Affektive Störungen
- ICD-10 F4: u.a. Neurotische und Somatoforme Störungen
- ICD-10 F5: u.a. Verhaltensauffälligkeiten mit körperlichen Störungen und Faktoren
- ICD-10 F6: u.a. Persönlichkeitsstörungen
- ICD-10 F7: u.a. Intelligenzminderung
- ICD-10 F8: u.a. Entwicklungsstörungen
- ICD-10 F9: u.a. Verhaltens- und emotionale Störungen mit Beginn in der Kindheit und Jugend

1. Wenn ja lt. Frage 24: Welche genetischen Testungen bei psychischen Störungen zur Bestimmung einer Diagnose führen Sie selbst durch oder haben diese angefordert?

|  |  | **ja** | **nein** |
| --- | --- | --- | --- |
|  | 1. Pharmako**genetische** Testungen (untersuchen den Einfluss der genetischen Ausstattungen auf Arzneimittelwirkungen) | 1 | 2 |
|  | 1. Pharmako**kinetische** genetische Testungen (Pharmakokinetik betrifft den Metabolismus der Medikamente, z.B. durch die Familie der CYP450 Enzyme. Es können die Gene dieser Enzyme untersucht werden) | 1 | 2 |
|  | 1. Pharmako**dynamische** genetische Testungen (untersuchen den Einfluss der genetischen Ausstattungen der Zielstrukturen der Medikamente, z.B. Neurotransmitter-Rezeptor-Gene, auf Arzneimittelwirkungen) | 1 | 2 |

1. Wenn ja lt. Frage 26a: Bei welchen Indikationen führen Sie pharamko**genetische** Testungen durch bzw. haben diese angefordert?

- ICD-10 F0: u.a. Organische Störungen
- ICD-10 F1: u.a. Psychische Störungen durch psychotrope Substanzen
- ICD-10 F2: u.a. Schizophrenien, schizotype und wahnhafte Störungen
- ICD-10 F3: u.a. Affektive Störungen
- ICD-10 F4: u.a. Neurotische und Somatoforme Störungen
- ICD-10 F5: u.a. Verhaltensauffälligkeiten mit körperlichen Störungen und Faktoren
- ICD-10 F6: u.a. Persönlichkeitsstörungen
- ICD-10 F7: u.a. Intelligenzminderung
- ICD-10 F8: u.a. Entwicklungsstörungen
- ICD-10 F9: u.a. Verhaltens- und emotionale Störungen mit Beginn in der Kindheit und Jugend

1. Wenn ja lt. Frage 26b: Bei welchen Indikationen führen Sie pharmako**kinetische** genetische Testungen durch bzw. haben diese angefordert?

- ICD-10 F0: u.a. Organische Störungen
- ICD-10 F1: u.a. Psychische Störungen durch psychotrope Substanzen
- ICD-10 F2: u.a. Schizophrenien, schizotype und wahnhafte Störungen
- ICD-10 F3: u.a. Affektive Störungen
- ICD-10 F4: u.a. Neurotische und Somatoforme Störungen
- ICD-10 F5: u.a. Verhaltensauffälligkeiten mit körperlichen Störungen und Faktoren
- ICD-10 F6: u.a. Persönlichkeitsstörungen
- ICD-10 F7: u.a. Intelligenzminderung
- ICD-10 F8: u.a. Entwicklungsstörungen
- ICD-10 F9: u.a. Verhaltens- und emotionale Störungen mit Beginn in der Kindheit und Jugend

1. Wenn ja lt. Frage 26c: Bei welchen Indikationen führen Sie pharmko**dynamische** genetische Testungen durch bzw. haben diese angefordert?

- ICD-10 F0: u.a. Organische Störungen
- ICD-10 F1: u.a. Psychische Störungen durch psychotrope Substanzen
- ICD-10 F2: u.a. Schizophrenien, schizotype und wahnhafte Störungen
- ICD-10 F3: u.a. Affektive Störungen
- ICD-10 F4: u.a. Neurotische und Somatoforme Störungen
- ICD-10 F5: u.a. Verhaltensauffälligkeiten mit körperlichen Störungen und Faktoren
- ICD-10 F6: u.a. Persönlichkeitsstörungen
- ICD-10 F7: u.a. Intelligenzminderung
- ICD-10 F8: u.a. Entwicklungsstörungen
- ICD-10 F9: u.a. Verhaltens- und emotionale Störungen mit Beginn in der Kindheit und Jugend

1. Wenn ja lt. Frage 24 bzw. ja lt. Frage 26a, b, c: Wie oft haben Sie in den letzten 10 Jahren…..bei psychischen Störungen gemacht oder angefordert?

|  |  | **1-10mal** | **11-20mal** | **Mehr als 20mal** |
| --- | --- | --- | --- | --- |
|  | 1. Genetische Testungen | 1 | 2 | 3 |
|  | 1. Pharmako**genetische** Testungen (untersuchen den Einfluss der genetischen Ausstattungen auf Arzneimittelwirkungen) | 1 | 2 | 3 |
|  | 1. Pharmako**kinetische** genetische Testungen (Pharmakokinetik betrifft den Metabolismus der Medikamente, z.B. durch die Familie der CYP450 Enzyme. Es können die Gene dieser Enzyme untersucht werden) | 1 | 2 | 3 |
|  | 1. Pharmako**dynamische** genetische Testungen (untersuchen den Einfluss der genetischen Ausstattungen der Zielstrukturen der Medikamente, z.B. Neurotransmitter-Rezeptor-Gene, auf Arzneimittelwirkungen) | 1 | 2 | 3 |

1. Wenn ja lt. Frage 26b: Bei welchen Psychopharmaka haben Sie pharmako**kinetische** genetische Testungen gemacht oder angefordert? (Mehrfachnennungen möglich)

- Antidepressiva
- Antipsychotika
- Phasenprophylaktika
- Anxiolytika
- Andere und zwar: ___________________

1. Wenn ja lt. Frage 26c: Bei welchen Psychopharmaka haben Sie pharmako**dynamische** genetische Testungen gemacht oder angefordert? (Mehrfachnennungen möglich)

- Antidepressiva
- Antipsychotika
- Phasenprophylaktika
- Anxiolytika
- Andere und zwar: ___________________

1. Wenn ja lt. Frage 24: Bei wem haben Sie genetische Testungen zur Bestimmung einer Diagnose gemacht oder angeordnet? (Mehrfachnennungen möglich)

- Frauen
- Männer
- Kinder und Jugendliche

1. Wenn ja lt. Frage 26b: Bei wem haben Sie pharmako**kinetische** genetische Testungen bei psychischen Störungen gemacht oder angeordnet? (Mehrfachnennungen möglich)

- Frauen
- Männer
- Kinder und Jugendliche

1. Wenn ja lt. Frage 26c: Bei wem haben Sie pharmako**dynamische** genetische Testungen bei psychischen Störungen gemacht oder angeordnet? (Mehrfachnennungen möglich)

- Frauen
- Männer
- Kinder und Jugendliche

1. Wenn ja lt. Frage 24 Werden genetische Testungen bei psychischen Störungen von den Sozialversicherungen ausreichend abgegolten?

1 ja

2 nein

Abschließend noch ein paar Angaben zu Ihrer Person:

A. Geschlecht:

1 männlich

2 weiblich

B. Alter: ________ Jahre (genau eintragen!)

1 bis 35 Jahre

2 bis 55 Jahre

3 älter als 55 Jahre

C. Fachärzt*innenrichtung (Mehrfachnennungen möglich):

- Humangenetik
- Kinder- und Jugendneuropsychiatrie
- Kinder- und Jugendpsychiatrie
- Kinder- und Jugendpsychiatrie und Psychotherapeutische Medizin
- Medizinische Genetik
- Neurologie
- Neurologie und Psychiatrie
- Psychiatrie
- Psychiatrie und Neurologie
- Psychiatrie und Psychotherapeutische Medizin
- Anderes und zwar welches Fach: _______________________________

D. Haben Sie ein Zusatzgebiet erworben?

1 ja

2 nein

E. Wenn ja, welches?

1 Humangenetik

2 Kinder- und Jugendpsychiatrie

F. Wo sind Sie tätig?

1 Ordination mit Kassenverträgen

2 Ordination als Wahlarzt/Wahlärztin

3 Krankenhaus

4 Psychosozialer Dienst

5 Andere Einrichtung und zwar: _________________________

G. Haben Sie ein ÖAK Diplom Genetik?

1 ja

2 nein
