# Supplementary material for: A survey in Austria supports the significance of genetic counseling and pharmacogenetic testing for mental illness
Source: Front Psychiatry. 2024 Oct 3;15:1436875. doi: 10.3389/fpsyt.2024.1436875 (PMC11484073; doi:10.3389/fpsyt.2024.1436875)
Supplement: Supplementary file 1 [file DataSheet1.zip › Appendix 3.DOCX]

Appendix 3.

**Questionnaire “Genetic counseling and testing from the perspective of the Austrian population”**

In today's survey we are focusing on a medical topic. It's not about you knowing specific things, but about your personal emotional assessment.

1. Have you ever been diagnosed with a mental disorder?

1 yes

2 no

1. If so, according to question 1: which one? (multiple answers possible)

- dementia
- problematic alcohol consumption
- problematic use of substances (except alcohol)
- schizophrenia or psychosis
- depression
- bipolar disorder
- anxiety disorder
- posttraumatic stress disorder
- eating disorder
- personality disorder
- other

1. How useful do you think genetic counseling* is for mental disorders?

Please use a scale of 1-5 for your assessment, where

1 means very useful,

5 means not useful at all.

You can grade in between.

| Very useful | 1 | 2 | 3 | 4 | 5 | Not useful at all |
| --- | --- | --- | --- | --- | --- | --- |

*Genetic counseling is a medical consultation that informs patients about any genetic disorders that may exist and the resulting diseases or risks

1. How useful do you think genetic counseling is for physical illnesses?

Please use a scale of 1-5 for your assessment, where

1 means very useful,

5 means not useful at all.

You can grade in between.

| Very useful | 1 | 2 | 3 | 4 | 5 | Not useful at all |
| --- | --- | --- | --- | --- | --- | --- |

1. Would you get genetic counseling if you needed it (because of a mental disorder)?

1 yes

2 no

1. If so, according to question 5: in what context? (Multiple answers possible)

- with your own mental illness
- in the case of mental illness of family members
- if you want to have children
- when choosing a partner
- other

1. Have you already taken advantage of genetic counseling for mental disorder (because of your own conspicuous features or conspicuous features in members of the family)?

1 yes

2 no

1. If yes, according to question 7: what mental illness or problems? (multiple answers possible) (yourself or in your family environment)

- dementia
- problematic alcohol consumption
- problematic use of substances (except alcohol)
- schizophrenia or psychosis
- depression
- bipolar disorder
- anxiety disorder
- posttraumatic stress disorder
- eating disorder
- personality disorder
- suicides in the family
- other

1. Who would you go to if you wanted genetic counseling?

- family doctor
- specialist depending on the diagnosis
- hospital
- institute of Human Genetics
- other

1. Should genetic counseling in Austria be offered by a professional group with their own special training?

- yes, through professional group (“genetic counselor”)
- no, it should be done by a medical doctor

1. Have you ever had genetic testing** done?

1 yes

2 no

**Genetic testing refers to genetic examinations (e.g. DNA analyses) to determine or improve the diagnosis or therapy)

1. If so, according to question 11: in what context? (multiple answers possible)

- in case of mental illness
- in case of physical illness
- to choose the right therapy for me when I'm sick / to determine the "right" therapeutic approach
- other

1. If you were offered a genetic test, you would do it?

1 yes

2 no

1. If not, according to question 13: why not? (multiple answers possible)

- because of fear of the results of the test
- because of fear of being stigmatized if I tell someone (that I took the test or have an illness)
- because if I were diagnosed with an illness, I would feel guilty that I could inherit this illness
- because I expect problems with the insurance
- because I expect problems with the employer
- because I expect problems with family
- because I expect problems with friends
- because I don't see the need
- other

1. Do you think that genetic testing has negative consequences for mental illnesses?

1 yes

2 no

1. If so, according to question 15: in what respects? (multiple answers possible)

- significant increase in fear of the test result
- increased concern about being stigmatized if I tell someone (that I took the test or have a disease or am at risk for a disease)
- high levels of guilt when I find out that I could inherit the disease
- problems with insurance
- problems with the employer
- problems with family
- problems with friends
- other

1. Do you believe that genetic testing in mental illnesses has positive effects?

1 yes

2 no

1. If so, according to question 17: in what respects? (multiple answers possible)

- to be certain that I have or do not have the disease / to gain certainty
- inner peace of mind that I know
- in the event of illness, I receive better treatment, i.e. targeted, specific treatment that is tailored to my illness
- other

1. Would you share the results of a genetic test with other people?

1 yes

2 no

1. If so, according to question 19: to whom? (multiple answers possible)

- parents
- partner
- children
- siblings
- friends
- employer
- other

1. Should genetic testing for mental illnesses be covered by social security?

1 yes

2 no

1. Would you also pay privately for genetic testing for mental illnesses, i.e. cover the costs yourself?

1 yes

2 no

1. In your opinion, are genetic tests currently available that make it possible to diagnose mental disorders?

1 yes

2 no

1. If yes, according to question 23: Do the currently available genetic tests allow an assessment of a person's risk of developing a particular mental illness?

1 yes

2 no

1. How useful do you think genetic testing is for determining a diagnosis in the case of mental disorders?

Please use a scale of 1-5 for your assessment, where

1 means very useful,

5 means not useful at all.

You can grade in between.

| Very useful | 1 | 2 | 3 | 4 | 5 | Not useful at all |
| --- | --- | --- | --- | --- | --- | --- |

1. How useful do you think genetic testing is for physical illnesses to determine a diagnosis?

Please use a scale of 1-5 for your assessment, where

1 means very useful,

5 means not useful at all.

You can grade in between.

| Very useful | 1 | 2 | 3 | 4 | 5 | Not useful at all |
| --- | --- | --- | --- | --- | --- | --- |

1. Would it reassure you if you learned that genetic causes are responsible for your mental disorder?

1 yes

2 no

1. Do you think that discovering genetic causes of mental illness could help provide better, more specific treatment?

1 yes

2 no

1. Do you believe that genetic testing can help predict response to drug therapy for mental illness or select a medication that is right for you?

1 yes

2 no

1. Do you believe that genetic testing can help predict response to drug therapy for a physical illness or select a drug that is right for you?

1 yes

2 no

1. How likely would you be to get genetic testing if you have severe side effects to a medication?

Please use a scale of 1-5 for your assessment, where

1 means most likely,

5 means not likely at all.

You can grade in between.

| Most likely | 1 | 2 | 3 | 4 | 5 | Not likely at all |
| --- | --- | --- | --- | --- | --- | --- |

1. How likely is it you will be genetically tested if you do not respond to drug therapy?

Please use a scale of 1-5 for your assessment, where

1 means most likely,

5 means not likely at all.

You can grade in between.

| Most likely | 1 | 2 | 3 | 4 | 5 | Not likely at all |
| --- | --- | --- | --- | --- | --- | --- |

1. Have you ever requested genetic testing (regardless of the purpose) online

1 yes

2 no

1. If so, according to question 33: why?

- to determine the risk of disease
- to find out something about my ancestry/origin
- other

1. If yes, according to question 33: Have you discussed such a genetic test (requested via the internet) with a doctor?

1 yes

2 no
