# Supplementary material for: A survey in Austria supports the significance of genetic counseling and pharmacogenetic testing for mental illness
Source: Front Psychiatry. 2024 Oct 3;15:1436875. doi: 10.3389/fpsyt.2024.1436875 (PMC11484073; doi:10.3389/fpsyt.2024.1436875)
Supplement: Supplementary file 1 [file DataSheet1.zip › Appendix 4.DOCX]

Appendix 4.

**Questionnaire "Genetic counseling and testing from an expert perspective”**

Good day! My name is………...from the Austrian Gallup Institute.

We are currently conducting a study in the medical field and are interviewing specialists and experts about the situation of genetic counseling and testing in Austria.

The results of this study will be published and presented at scientific conferences.

The survey will take approximately 10 minutes.

Your information will of course be treated with absolute confidentiality and evaluated anonymously.

Can I ask you a few questions about this?

If yes, continue with the interview (either immediately or make an appointment).

If not, thank you and END.

#

1. How useful do you think genetic counseling is for mental disorders?

1 means: very useful

5 means: not useful at all

You can grade in between!

|  | Very useful | 1 | 2 | 3 | 4 | 5 | Not useful at all |
| --- | --- | --- | --- | --- | --- | --- | --- |

2. Do you provide genetic counseling for mental disorders yourself or have you provided genetic counseling for mental disorders yourself?

1 yes

2 no

3. If yes, according to question 2: How often have you provided genetic counseling for mental disorders in the last 10 years?

o 1-10 times

o 11-20 times

o more than 20 times

4. If yes, according to question 2: Whom have you given genetic counseling regarding mental disorders? (multiple answers possible)

1 patient

2 relatives

3 others, namely:_______________________

5. If yes, according to question 2: Which target groups did you provide genetic counseling? (multiple answers possible)

1 women

2 men

3 children and adolescents

6. Have you been asked for genetic counseling by patients after they had ordered genetic tests on the internet and received the result directly from the laboratory without counseling (“direct-to-consumer test”, DTC; excluding professionals)?

1 yes – how often has this happened? _____________(insert number, only 1 number)

2 no

7. If yes, according to question 2: For which mental disorders have you done genetic counseling? (multiple answers possible)

o ICD-10 F0: e.g. organic disorders

o ICD-10 F1: e.g. mental disorders caused by psychotropic substances

o ICD-10 F2: e.g. schizophrenia, schizotypal and delusional disorders

o ICD-10 F3: e.g. affective disorders

o ICD-10 F4: e.g. neurotic and somatoform disorders

o ICD-10 F5: e.g. behavioral problems with physical disorders and factors

o ICD-10 F6: e.g. personality disorders

o ICD-10 F7: e.g. intellectual disability

o ICD-10 F8: e.g. developmental disorders

o ICD-10 F9: e.g. behavioral and emotional disorders with onset in childhood and adolescence

8. If yes, according to question 2: Counseling based on…. (multiple answers possible)

1 family history

2 results of genetic tests

3 other, namely:____________________________

9. If yes, according to question 2: Have you referred patients for genetic testing for mental disorders before or after genetic counseling?

1 yes

2 no

10. Have you referred patients to specialized institutions, such as institutes for human genetics, before or after genetic counseling?

1 yes

2 no

ALL:

11. How satisfactory do you consider the genetic counseling training in Austria?

1 means: very satisfactory

5 means: not at all satisfactory

You can grade in between!

|  | Very satisfactory | 1 | 2 | 3 | 4 | 5 | Not at all satisfactory |
| --- | --- | --- | --- | --- | --- | --- | --- |

12. Have you undergone special training in genetic counseling (in Austria or abroad)?

1 yes – if yes, in what way? ________________________

2 no

13. Should genetic counseling in Austria be offered by a professional group with their own special training?

o yes, through professional group “Genetic Counselor”

o no, it should be done by a doctor

14. Are genetic tests currently available the use of which enables the diagnosis of mental disorders?

1 yes

2 no

15. If yes, according to question 14: For what indications?

o ICD-10 F0: e.g. organic disorders

o ICD-10 F1: e.g. mental disorders caused by psychotropic substances

o ICD-10 F2: e.g. schizophrenia, schizotypal and delusional disorders

o ICD-10 F3: e.g. affective disorders

o ICD-10 F4: e.g. neurotic and somatoform disorders

o ICD-10 F5: e.g. behavioral problems with physical disorders and factors

o ICD-10 F6: e.g. personality disorders

o ICD-10 F7: e.g. intellectual disability

o ICD-10 F8: e.g. developmental disorders

o ICD-10 F9: e.g. behavioral and emotional disorders with onset in childhood and adolescence

16. Are genetic tests currently available that can be used to predict the course of a mental disorder?

1 yes

2 no

17. If yes, according to question 16: For what indications?

o ICD-10 F0: e.g. organic disorders

o ICD-10 F1: e.g. mental disorders caused by psychotropic substances

o ICD-10 F2: e.g. schizophrenia, schizotypal and delusional disorders

o ICD-10 F3: e.g. affective disorders

o ICD-10 F4: e.g. neurotic and somatoform disorders

o ICD-10 F5: e.g. behavioral problems with physical disorders and factors

o ICD-10 F6: e.g. personality disorders

o ICD-10 F7: e.g. intellectual disability

o ICD-10 F8: e.g. developmental disorders

o ICD-10 F9: e.g. behavioral and emotional disorders with onset in childhood and adolescence

18. Do the currently available genetic tests allow an assessment of a person's risk of developing certain mental disorders?

1 yes

2 no

19. If yes, according to question 18: For what indications?

o ICD-10 F0: e.g. organic disorders

o ICD-10 F1: e.g. mental disorders caused by psychotropic substances

o ICD-10 F2: e.g. schizophrenia, schizotypal and delusional disorders

o ICD-10 F3: e.g. affective disorders

o ICD-10 F4: e.g. neurotic and somatoform disorders

o ICD-10 F5: e.g. behavioral problems with physical disorders and factors

o ICD-10 F6: e.g. personality disorders

o ICD-10 F7: e.g. intellectual disability

o ICD-10 F8: e.g. developmental disorders

o ICD-10 F9: e.g. behavioral and emotional disorders with onset in childhood and adolescence

20. How useful do you think genetic testing is for determining a diagnosis in the case of mental disorders?

1 means: very useful

5 means: not useful at all

You can grade in between!

|  | Very useful | 1 | 2 | 3 | 4 | 5 | Not useful at all |
| --- | --- | --- | --- | --- | --- | --- | --- |

21. Do you think genetic testing for mental disorders increases stigma and discrimination against mentally ill people?

1 yes

2 no

22. How useful do you think the following tests are for mental disorders?

1 means: very useful

5 means: not useful at all

You can grade in between!

|  | Very useful |  |  |  | Not useful at all |
| --- | --- | --- | --- | --- | --- |
| 1. Pharmaco**genetic** testing (examine the influence of genetic makeup on drug effects) | 1 | 2 | 3 | 4 | 5 |
| 1. Pharmaco**kinetic** genetic testing (Pharmacokinetics concerns the metabolism of drugs, e.g. through the family of CYP450 enzymes. The genes of these enzymes can be examined) | 1 | 2 | 3 | 4 | 5 |
| 1. Pharmaco**dynamic** genetic testing (examine the influence of the genetic makeup of the drug's target structures, e.g. neurotransmitter receptor genes, on drug effects) | 1 | 2 | 3 | 4 | 5 |

23. Do you have your own genetic testing laboratory?

1 yes

2 no

24. Do you carry out or have you requested genetic tests for mental disorders to determine a diagnosis?

1 yes

2 no

25. If yes, according to question 24: For what indications?

o ICD-10 F0: e.g. organic disorders

o ICD-10 F1: e.g. mental disorders caused by psychotropic substances

o ICD-10 F2: e.g. schizophrenia, schizotypal and delusional disorders

o ICD-10 F3: e.g. affective disorders

o ICD-10 F4: e.g. neurotic and somatoform disorders

o ICD-10 F5: e.g. behavioral problems with physical disorders and factors

o ICD-10 F6: e.g. personality disorders

o ICD-10 F7: e.g. intellectual disability

o ICD-10 F8: e.g. developmental disorders

o ICD-10 F9: e.g. behavioral and emotional disorders with onset in childhood and adolescence

26. If yes, according to question 24: Which genetic tests for mental disorders to determine a diagnosis do you carry out yourself or have you requested?

|  | yes | no |
| --- | --- | --- |
| a) Pharmaco**genetic** testing (examine the influence of genetic makeup on drug effects) | 1 | 2 |
| b) Pharmaco**kinetic** genetic testing (Pharmacokinetics concerns the metabolism of drugs, e.g. through the family of CYP450 enzymes. The genes of these enzymes can be examined) | 1 | 2 |
| c) Pharmaco**dynamic** genetic testing (examine the influence of the genetic makeup of the drug target structures, e.g. neurotransmitter receptor genes, on drug effects) | 1 | 2 |

27. If yes, according to question 26 a: For which indications do you carry out pharmaco**genetic** testing or have you requested it?

o ICD-10 F0: e.g. organic disorders

o ICD-10 F1: e.g. mental disorders caused by psychotropic substances

o ICD-10 F2: e.g. schizophrenia, schizotypal and delusional disorders

o ICD-10 F3: e.g. affective disorders

o ICD-10 F4: e.g. neurotic and somatoform disorders

o ICD-10 F5: e.g. behavioral problems with physical disorders and factors

o ICD-10 F6: e.g. personality disorders

o ICD-10 F7: e.g. intellectual disability

o ICD-10 F8: e.g. developmental disorders

o ICD-10 F9: e.g. behavioral and emotional disorders with onset in childhood and adolescence

28. If yes, according to question 26 b: For which indications do you carry out pharmaco**kinetic** genetic testing or have you requested it?

o ICD-10 F0: e.g. organic disorders

o ICD-10 F1: e.g. mental disorders caused by psychotropic substances

o ICD-10 F2: e.g. schizophrenia, schizotypal and delusional disorders

o ICD-10 F3: e.g. affective disorders

o ICD-10 F4: e.g. neurotic and somatoform disorders

o ICD-10 F5: e.g. behavioral problems with physical disorders and factors

o ICD-10 F6: e.g. personality disorders

o ICD-10 F7: e.g. intellectual disability

o ICD-10 F8: e.g. developmental disorders

o ICD-10 F9: e.g. behavioral and emotional disorders with onset in childhood and adolescence

29. If yes, according to question 26 c: For which indications do you carry out pharmaco**dynamic** genetic testing or have you requested it?

o ICD-10 F0: e.g. organic disorders

o ICD-10 F1: e.g. mental disorders caused by psychotropic substances

o ICD-10 F2: e.g. schizophrenia, schizotypal and delusional disorders

o ICD-10 F3: e.g. affective disorders

o ICD-10 F4: e.g. neurotic and somatoform disorders

o ICD-10 F5: e.g. behavioral problems with physical disorders and factors

o ICD-10 F6: e.g. personality disorders

o ICD-10 F7: e.g. intellectual disability

o ICD-10 F8: e.g. developmental disorders

o ICD-10 F9: e.g. behavioral and emotional disorders with onset in childhood and adolescence

30. If yes, according to question 24 or yes, according to question 26 a, b, c: How often in the last 10 years have you done or requested… for mental disorders?

|  | 1-10 times | 11-20 times | More than 20 times |
| --- | --- | --- | --- |
| a) Genetic testing | 1 | 2 | 3 |
| b) Pharmaco**genetic** testing (examine the influence of genetic makeup on drug effects) | 1 | 2 | 3 |
| c) Pharmaco**kinetic** genetic testing (Pharmacokinetics concerns the metabolism of drugs, e.g. through the family of CYP450 enzymes. The genes of these enzymes can be examined) | 1 | 2 | 3 |
| d) Pharmaco**dynamic** genetic testing (examine the influence of the genetic makeup of the drug target structures, e.g. neurotransmitter receptor genes, on drug effects) | 1 | 2 | 3 |

31. If yes, according to question 26 b: For which psychotropic drugs have you carried out or requested pharmaco**kinetic** genetic testing? (multiple answers possible)

o antidepressants

o antipsychotics

o mood stabilizers

o anxiolytics

o others namely: ___________________

32. If yes, according to question 26 c: For which psychotropic drugs have you carried out or requested pharmaco**dynamic** genetic testing? (multiple answers possible)

o antidepressants

o antipsychotics

o mood stabilizers

o anxiolytics

o others namely: ___________________

33. If yes, according to question 24: For whom did you carry out or order genetic testing to determine a diagnosis? (multiple answers possible)

o women

o men

o children and adolescents

34. If yes, according to question 26 b: For whom have you carried out or ordered pharmaco**kinetic** genetic testing for mental disorders? (multiple answers possible)

o women

o men

o children and adolescents

35. If yes, according to question 26 c: For whom have you carried out or ordered pharmaco**dynamic** genetic testing for mental disorders? (multiple answers possible)

o women

o men

o children and adolescents

36. If yes, according to question 24: Are genetic tests for mental disorders sufficiently covered by social insurance?

1 yes

2 no
